# Virulence role of the outer membrane protein CarO in carbapenem-resistant *Acinetobacter baumannii*

**DOI:** 10.1080/21505594.2020.1855912

**Published:** 2020-12-10

**Authors:** Gema Labrador-Herrera, Antonio J. Pérez-Pulido, Rocío Álvarez-Marín, Carlos S. Casimiro-Soriguer, Tania Cebrero-Cangueiro, Jorgelina Morán-Barrio, Jerónimo Pachón, Alejandro M. Viale, María Eugenia Pachón-Ibáñez

**Affiliations:** a Clinical Unit of Infectious Diseases, Microbiology, and Preventive Medicine, University Hospital Virgen del Rocío, Seville, Spain; b Institute of Biomedicine of Seville (IBiS), University of Seville/CSIC/University Hospital Virgen del Rocío, Seville, Spain; c Andalusian Centre for Developmental Biology (CABD, UPO-CSIC-JA), Faculty of Experimental Sciences (Genetics Area), Pablo de Olavide University, Seville, Spain; d Department of Medicine, University of Seville, Seville, Spain; e Instituto de Biología Molecular y Celular de Rosario (IBR), Departamento de Microbiología, Facultad de Ciencias Bioquímicas y Farmacéuticas, CONICET, Universidad Nacional de Rosario (UNR), Rosario, Argentina

**Keywords:** *Acinetobacter baumannii*, bloodstream infections, carbapenem-resistance, whole-genome sequencing, virulence factor, CarO

## Abstract

Novel approaches to treat carbapenem-resistant *Acinetobacter baumannii* (CRAB) infections are urgently needed and anti-virulence drugs represent promising alternatives, but our knowledge on potential targets is scarce. We searched for potential *A. baumannii* virulence factors by whole-genome sequencing-based comparisons of CRAB clinical isolates causing bloodstream infections secondary to ventilator-associated pneumonia from demographics and clinically homogeneous patients, who received optimal treatment but with different clinical outcomes. Thus, the *carO* gene was interrupted in CRAB isolates from surviving patients, while it was intact in isolates from non-surviving patients, and proteomic/immunoblot techniques corroborated it. When the virulence role of *A. baumannii* CarO was analyzed in model systems, isogenic Δ*carO* mutants and a CRAB clinical isolate with truncated CarO, showed lower ability to adhere and invade A549 cells and *in vivo* virulence. This unnoticed virulence role for CarO postulate this *A. baumannii* outer membrane protein as a potential target for new therapies against CRAB infections.

## Introduction

*Acinetobacter baumannii* is one of the most successful opportunistic pathogens responsible for healthcare-acquired infections worldwide [1]. Of particular importance is its ability to cause bloodstream infections (BSI) in critically ill patients, mainly due to ventilated associated pneumonia (VAP), associated with septic shock [2]. Crude mortality rates in these patients have been reported between 30% and 76% [3]. This pathogen is endowed with an extraordinary capability to develop resistance to antibiotics, including carbapenems [1–3]. This situation has prompted the search of new therapeutic strategies to deal with carbapenem-resistant *A. baumannii* (CRAB) infections, and non-antimicrobial approaches targeting bacterial virulence factors might represent promising alternatives [4]. Nevertheless, the knowledge on *A. baumannii* virulence traits is relatively scarce [5]. Whole-genome sequencing (WGS) comparisons between different strains of a given pathogen causing infections are employed for the identification of potential virulence factors [6]. However, the origin of the strains and the patients’ clinical characteristics should be carefully considered when drawing conclusions on possible virulence factors, or these might not be definite [7–9].

This study aimed to gain insight, using WGS complemented with proteomic and immunoblot analyses, and *in vitro* and *in vivo* model systems, into putative *A. baumannii* virulence factors associated with the different outcomes of clinically homogeneous patients with CRAB BSI secondary to VAP.

## Materials and methods

### Clinical isolates and clinical and demographic characteristics of the patients

Six CRAB clinical isolates (Supplementary Table S1) from blood cultures of six patients with BSI secondary to VAP were used. These patients were selected from a cohort of adult patients with VAP admitted to the intensive care unit (ICU) of a tertiary hospital, 15 of them with secondary BSI [10]. Patients’ clinical and demographic characteristics and isolate codes are detailed in Table 1. The criteria to select them were: i) isolation from young adult patients without underlying chronic comorbidities (Charlson index = 0), acute physiology and chronic health evaluation (APACHE II) ≥15, similar clinical pulmonary infection score (CPIS) at the inclusion, and no co-infections during hospital stay; ii) appropriate antimicrobial treatment of the VAP [10], being all the isolates colistin-susceptible (MIC range: 0.03–0.125 µg/mL) and without colistin-heteroresistance; and iii) same pulsed-field gel electrophoresis pattern as their corresponding VAP CRAB isolates, confirming the source of BSI. Three patients died (patients 1–3, infected by B1, B4, and B7 *A. baumannii* isolates, respectively) and three survived (patients 4–6, infected by B8, B9, and B11 *A. baumannii* isolates, respectively). From now on we will refer to them as “both groups of clinical isolates.”

**Table 1.** Patients’ demographics, clinical features, and antibiotic therapy

	Patient 1	Patient 2	Patient 3	Patient 4	Patient 5	Patient 6
BSI CRAB isolates (codes)	B1	B4	B7	B8	B9	B11
Age (years)	34	48	41	35	48	26
Sex	Female	Male	Female	Male	Male	Male
Charlson index^a^	0	0	0	0	0	0
Diagnosis at ICU admission	CAP^b^	Sepsis	Stroke	Abdominal surgery	Fournier’s gangrene	Cardiovascular surgery
APACHE II score^c^	23	20	20	15	17	23
CPIS score^d^	8	6	8	7	6	6
Pitt score^e^	6	12	6	7	6	3
Co-infection	No	No	No	No	No	No
Septic shock	Yes	Yes	Yes	Yes	No	Yes
Acute renal failure	Yes	No	Yes	Yes	No	Yes
Multiorgan failure	Yes	No	Yes	No	No	Yes
CMS^f^ treatment (MIU^g^/24 h)	6	8	6	9	9	6
ICU length-of stay (days)	23	18	30	46	31	20
30-days mortality	Yes	Yes	Yes	No	No	No

^a^Charlson: comorbidity index; ^b^CAP: community-acquired pneumonia; ^c^APACHE II: acute physiology and chronic health evaluation II score; ^d^CPIS: clinical pulmonary infection score; ^e^Pitt: severity bacteremia score; ^f^CMS: colistimethate sodium; ^g^MIU: million international units.

### DNA extraction, whole-genome sequencing and protein-coding gene annotation

DNA of the six CRAB clinical isolates was extracted using QIAamp® DNA Mini Kit. Sequencing was performed using MiSeq platform (Illumina), according to the standard protocol for WGS paired-end, producing 2 × 300 bp fragment reads. SPAdes v3.5 was chosen for *de novo* assembly of the reads into contigs [11]. Then, protein-coding genes were predicted by Prodigal v2.6.3, and the derived amino acid sequences were obtained [12]. The predicted proteins were functionally annotated by Sma3s v2 using the bacterial taxonomic division of UniProt database [13].

### Multilocus sequence typing and identification of known resistance and virulence mechanisms

Multilocus sequence typing (MLST) (Pasteur and Oxford MLST schemes) was performed by uploading the contigs files obtained from the *de novo* assembly of the WGS data of each of the six CRAB clinical isolates to the MLST web server v2.0 from the Center for Genomic Epidemiology (CGE) [14]. Then, several strategies were used to identify resistance mechanisms using the assembled WGS data from the six CRAB clinical isolates. Thus, ResFinder web server v3.2 from CGE [15] was used for acquired antimicrobial resistance genes, functional annotations including either the term “resistance” or “antibiotic” for other resistance genes, BLASTP [16] for point mutations in genes associated with quinolone and rifampicin resistance, and a combination of ISfinder [17], BLASTN searching on *Acinetobacter* sequence databases [16], and visual inspection for a detailed identification of insertion sequences (IS) located around *bla*_OXA_ genes on the sequence data derived from the WGS analysis of the clinical isolates described here (Supplementary Text S1). Secondly, two different approaches were used for the identification of known virulence genes, both based on the Virulence Factors of Pathogenic Bacteria database (VFDB) [18] (Supplementary Text S1).

### Differential protein-coding gene analyses

The amino acid sequences of all the predicted protein-coding genes of the six CRAB clinical isolates were clustered using the CD-HIT tool [19] with an identity threshold and coverage for the longer sequence of 95%. It brings together the same protein from the different isolates in the same cluster. So, if a specific cluster has no sequences coming from a specific isolate, it will be considered that this isolate lacks the gene coding for that protein. To determine protein variants between the isolates, a more specific protocol was designed using the standalone version of BLASTP [16] with identity threshold and query coverage of 100%. So, proteins from each isolate were independently compared to the proteins of the rest of isolates to discover variants. When a sequence showed or identity or query coverage between isolates different to 100% it was considered a differential protein-coding gene.

### CarO sequence alignments and analysis

The amino acid alignments of the inferred CarO proteins from the six CRAB clinical isolates were performed using MAFFT v7.312 with default setting [20]. When possible, the CarO protein sequence of a given isolate was assigned to a variant following the proposal of Mussi *et al*. [21]. The secondary structure of CarO was obtained from the Protein Data Bank (PDB) entry 4fuv.1.A, which presents an identical amino acid sequence. The multiple alignment with all data was depicted using Jalview v2.11.0 [22].

### Outer membrane protein profiles and immunoblotting

The outer membrane (OM) fractions from the six CRAB clinical isolates were isolated as described previously [23]. The outer membrane protein (OMP) profiles were determined by sodium dodecyl sulfate polyacrylamide gel electrophoresis (SDS-PAGE) using 4–15% SDS gels and the equivalent to 10 µg of total OM protein per sample, followed by SimplyBlue SafeStain staining. The differential bands found between both groups of clinical isolates were analyzed by liquid chromatography-tandem mass spectrometry (LC-MSMS). Proteins were identified using Proteome Discoverer v2.1 software, and results were filtered by a false discovery rate of 1% [24]. Western blot analysis was conducted after by incubating the polyvinylidene difluoride membrane with polyclonal antibodies against *A. baumannii* CarO elicited in rabbits [25], and peroxidase-labeled anti-rabbit immunoglobulin G (IgG) antibody from donkeys [26].

### Strains and isolates selected as model systems

**Figure 1.**
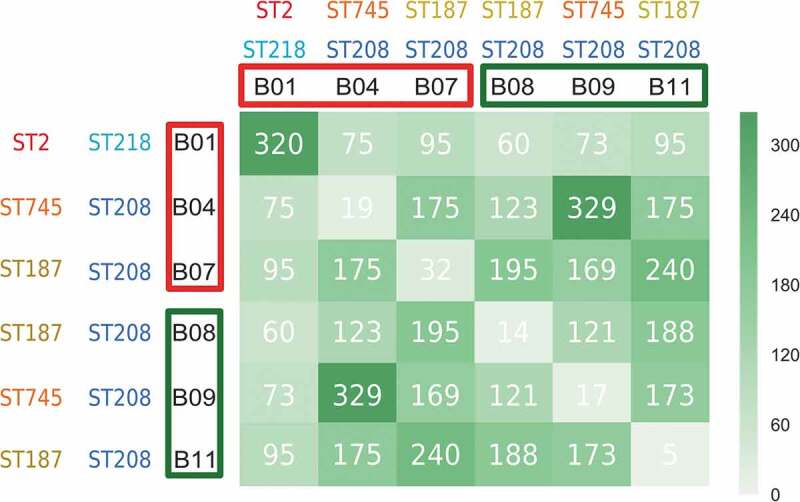
Graphical representation of exclusive protein-coding genes between pairs of CRAB clinical isolates. The records in the matrix represent the number of exclusive protein-coding genes between compared isolates (e. g., 320 is the number of exclusive protein-coding genes in the B1-B1 couple (i. e., B1 compared with itself), so no isolate other than B1 has these 320 protein-coding genes). The higher the number of exclusively shared protein-coding genes between compared isolates, the darker the green of the matrix cell. Isolates from non-surviving patients and surviving patients are highlighted in red and green colors, respectively. The corresponding STs following the Pasteur and Oxford schemes are also shown

The model strains ATCC 17978 and ATCC 19606 (type strain) were selected to analyze the role of CarO as a virulence factor in *A. baumannii*, as well as their corresponding mutants lacking CarO (Δ*carO*) and complemented strains with CarO expression restored (Δ*carO*/pWH1266-*carO*, with variants CarOIV and CarOI, respectively) [21]. Both wild-type (wt) strains and their Δ*carO* mutants were also transformed with the “empty” vector pWH1266 (wt/pWH1266 and Δ*carO*/pWH1266, respectively) and used as controls. Additionally, the two clinical isolates with the highest number of common genes (Figure 1), but with different CarO expression and isolated from patients with different outcome (B4 and B9), were used. Isolates/strains were grown for 18–20 h in Mueller-Hinton broth (MHB) at 37°C and 180 rpm. Kanamycin (20 µg/mL) and ticarcillin (80 µg/mL) were used to select Δ*carO* strains and strains harboring pWH1266 or pWH1266-*carO*, respectively. The cultured isolates/strains were collected by centrifugation (15 min at 4,500 g), rinsed with sterilized phosphate-buffered saline (PBS), and resuspended in Dulbecco’s modified Eagle’s medium (DMEM) before use in epithelial cell infection assays. All strains/isolates exhibited similar growth rates in Mueller-Hinton broth (MHB) (Supplementary Text S1 and Figure S1).

### A. baumannii adherence and invasion of cultured human lung epithelial cells

Human lung epithelial cells, line A549 (ATCC® CCL-185™), were seeded (10^5^ cells/well) for 24 h in 24-well plates. Before infection, cells were rinsed twice with PBS and then incubated with a 1:1000 dilution of an overnight culture of all the strains and isolates described above. Bacterial adherence and invasion assays were performed as previously described [26]. Assays were performed thrice in different days.

### Peritoneal sepsis murine model

Experiments were approved by the Committee on the Ethics of Animal Experiments of the University Hospital of Virgen del Rocío, Seville, Spain, and from the Ministry of *Agricultura, Pesca y Desarrollo Rural* (2012PI/246). A peritoneal sepsis murine model using 7–9-week-old immunocompetent C57BL/6 J female mice (approximately 18–20 g) [27] was used.

#### Determination of minimum lethal doses

Briefly, mice were inoculated intraperitoneally (i.p.) with 0.5 mL of bacterial dilutions (of 17978 wt, 17978 Δ*carO*, B4, and B9) mixed 1:1 with a saline solution of porcine stomach mucin (type II) at 10% (w/v), starting from an inoculum of 1.0 log_10_ CFU/mL approximately and ending with the first inoculum that cause 100% mice mortality (achievement of minimum lethal dose (MLD)). Groups of 6 mice per inoculum were used for 17978 cells, and groups of 4 mice for B4 and B9 clinical isolates, and survival rates were monitored during 7 days. With the 17978 Δ*carO*/pWH1266-*carO* complemented strain, only the inoculum used for the *in vivo* dissemination study (3.2 log_10_ CFU/mL) was tested, following the 3Rs principles (https://www.nc3rs.org.uk/the-3rs).

#### In vivo dissemination

Three groups of 14 mice each were inoculated i.p. with 0.5 mL of 3.2 log_10_ CFU/mL of 17978 wt, 17978 Δ*carO*, or 17978 Δ*carO*/pWH1266-*carO* strains. Eight- and twenty-four-hours post-inoculation, 7 animals of each group were randomly selected and sacrificed (200 µL sodium thiopental, i.p.). Then, bacterial loads were quantified in tissues (log_10_ CFU/g) and fluids (log_10_ CFU/mL).

### Statistical analysis

GraphPad Prism 6 (GraphPad-Software) was used. Student’s t-test and Mann–Whitney test were used to compare variables normally and not normally distributed, respectively. Survival curves and log-rank test were used to compare the survival distributions of mice. Significance was established at a *P*-value <0.05. Error bars on graphs represent the standard error of the mean (SEM).

## Results

### Multilocus sequence typing characterization of the carbapenem-resistant Acinetobacter baumannii clinical isolates

Oxford MLST scheme assigned ST218 for B1 and ST208 for the other isolates. Following the current definition of clonal complex [28], all six isolates were thus assigned to CC92 in the Oxford scheme. Pasteur MLST scheme, assigned ST2 for B1, ST745 for B4 and B9, and ST187 for B7, B8, and B11 isolates. Therefore, all six CRAB isolates were thus assigned to CC2 in the Pasteur scheme. It follows that all six isolates belong to CC92 (Oxford)/CC2 (Pasteur), the largest and most widely distributed *A. baumannii* global clone [28].

### Identification of known resistance and virulence mechanisms

All resistance and virulence mechanisms found in the six CRAB clinical isolates are described in the Supplementary Data sets S1 and S2, respectively. Carbapenem resistance could be attributed to the presence of different carbapenem-hydrolyzing class D β-lactamases [29] such as OXA-40/24 and OXA-109 (an OXA-51-type chromosomally encoded enzyme) in B1, and OXA-58 and OXA-66 (another OXA-51-type enzyme) in the other five clinical isolates, some of which were overexpressed due to the insertion of particular ISs upstream their genes (Supplementary Text S2).

### Differential presence of genes between carbapenem-resistant Acinetobacter baumannii clinical isolates from non-surviving and surviving patients

WGS comparisons indicated that all CRAB clinical isolates were different (Figure 1). When comparisons were performed between both groups of clinical isolates, some differences in a few number of genomic loci were found. The most notable difference between both groups of clinical isolates was noted at the *carO* locus encoding the OMP CarO. While the *carO* genes present in the isolates B1, B4 and B7 from the non-surviving patients were intact, those of the B8, B9, and B11 isolates from the surviving patients were found to be prematurely interrupted by different mutational events (see below). Other differences between isolates involved loci corresponding to bacteriophage genes, and implied small changes in length either at the N-terminus or C-terminus of the encoded proteins, or disruptions in the case of the B1 isolate. In addition, differences in length were also observed in the gene encoding the giant biofilm-associated protein Bap. However, the multiple arrays featuring immunoglobulin-like motifs common to this protein [30] made difficult the accurate assembly of the sequence data obtained for the different isolates, and whether significant differences exist at these loci between both groups of isolates is uncertain. A similar situation occurred for the locus encoding the pilus assembly protein FilE, which was found in both groups of isolates to encode for a variable number of repeats of the tripeptide TAP in the different isolates (data not shown).

### CarO sequence characterization

**Figure 2.**
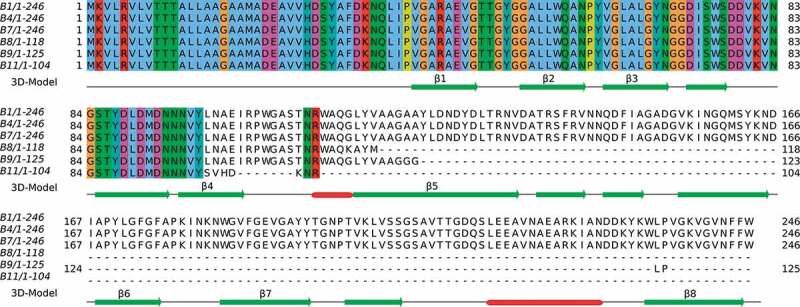
Alignment of CarO amino acids sequences from the six CRAB clinical isolates. The numbers represent the positions of amino acids. CarO protein was intact in the isolates from non-surviving patients (B1, B4, and B7), while it was truncated in those from surviving patients (B8, B9, and B11). The secondary structure obtained from the PDB structure 4fuv.1.A is shown in the 3D-Model track: α-helices in red color, and β-strands in green color. The eight strands from the CarO β-barrel have been tagged (β1-β8)

CarO protein sequences (complete and defective) inferred from the WGS data of the six CRAB clinical isolates and their corresponding alignments are shown in Figure 2. As noted above, complete *carO* genes each encoding the same protein of 246 amino acids were found in the isolates from the non-surviving patients (B1, B4, and B7). In addition, the comparison of the amino acid sequences of these CarO proteins with the four allelic variants present in the *A. baumannii* population [21] indicated that these isolates carried the same variant, designated CarOIII (GenBank accession number ABC46545.1). In contrast, the *carO* genes in isolates B8, B9, and B11 from the surviving patients were all prematurely interrupted by separate mutational events. Isolate B8 showed a 2-bp insertion in the *carO* gene introducing a frame-shift resulting in a premature stop codon and a putative truncated protein of only 118 amino acids long, isolate B9 a 1-bp insertion resulting in a putative truncated protein of only 125 amino acids long, and in isolate B11 an IS*Aba1* insertion was detected inside *carO* resulting in a putative truncated protein of only 104 amino acids long. Therefore, from the eight antiparallel β-strands that conform the β-barrel protein CarO, as judged by crystallographic analysis [31], isolates from non-surviving patients would generate, at best, only truncated CarO proteins ending shortly after the β-strand 4 (Figure 2).

### Outer membrane protein profiles of carbapenem-resistant Acinetobacter baumannii clinical isolates and detection of CarO

**Figure 3.**
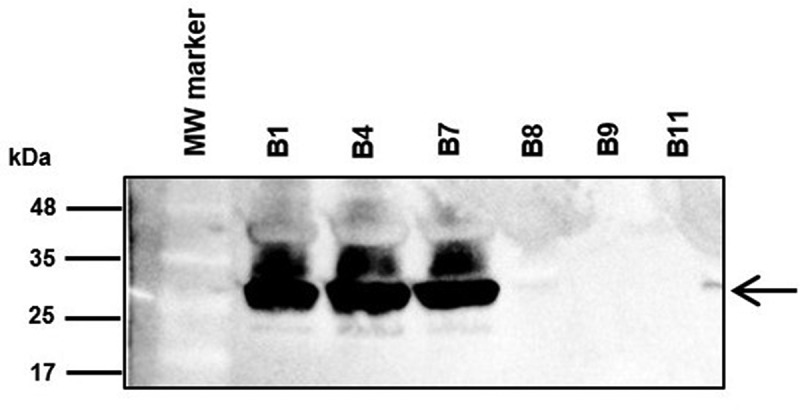
Immunodetection of CarO in outer membrane protein profiles. Outer membrane fractions were extracted from the bacteremic CRAB clinical isolates from non-surviving patients (B1, B4, and B7) and from surviving patients (B8, B9, and B11), and subjected to sodium dodecyl sulfate polyacrylamide gel electrophoresis (SDS-PAGE), followed by immunoblotting with polyclonal rabbit antibodies toward *A. baumannii* CarO. Molecular weight standards (kDa) are shown on the left. Black arrow on the right indicate the final position of CarO. MW, molecular weight

In agreement with the WGS analysis of the CRAB clinical isolates, a ~ 29 kDa protein band corresponding to the expected migration of CarO [25] was observed only in the isolates from the non-surviving patients by SDS-PAGE, and LC-MSMS analysis confirmed the identity of this band as CarO (data not shown). Western blot analysis confirmed these results and failed to indicate the presence of CarO in isolates from the surviving patients (Figure 3).

### Effect of CarO loss on A. baumannii adherence and invasion of human lung epithelial cells

**Figure 4.**
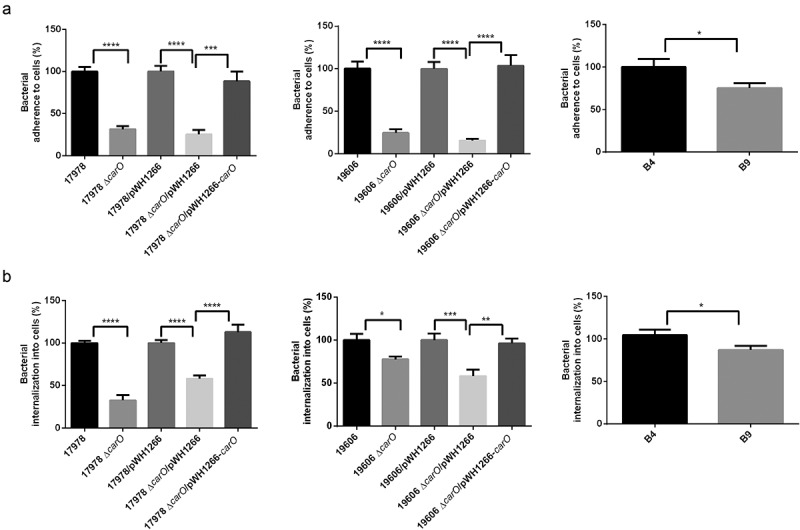
*A. baumannii* adherence and invasion into human lung A549 cells. A549 cell cultures were incubated with two reference *A. baumannii* strains: ATCC 17978 and ATCC 19606 wild-type (17978 and 19606); their isogenic *carO* deletion mutants (17978 Δ*carO* and 19606 Δ*carO*); both 17978 wt and 19606 wt with empty plasmid pWH1266 as controls (17978/pWH1266 and 19606/pWH1266); both 17978 Δ*carO* and 19606 Δ*carO* mutants with empty plasmid as controls (17978 Δ*carO*/pWH1266 and 19606 Δ*carO*/pWH1266); 17978 Δ*carO* and 19606 Δ*carO* mutants with plasmid pWH1266-*carO* expressing CarO (17978 Δ*carO*/pWH1266-*carO* and 19606 Δ*carO*/pWH1266-*carO*); and with the two CRAB clinical isolates B4 and B9. The percentages of bacterial adherence (a) and invasion (b) were subsequently measured. Data are represented as mean ± SEM (n = 3 replicates in different days). *P < 0.05, ** *P* < 0.01, *** *P* < 0.001, **** *P* < 0.0001, Student’s t-test and Mann-Whitney test

The loss of CarO reduced the adherence of 17978 wt and 19606 wt cells to A549 human lung epithelial cells (Figure 4(a)). Moreover, adherence was recovered when CarO expression in the Δ*carO* mutants was restored (Figure 4(a)). Similarly, the loss of *carO* reduced invasion of A549 cells by 17978 wt and 19606 wt cells (Figure 4(b)). Again, complementation of Δ*carO* mutants restored invasion to the levels of their parental wt strains (Figure 4(b)). In addition, the clinical isolate with a truncated *carO* gene (B9) exhibited lower adherence to A549 cells than the one with a functional CarO (B4) (Figure 4(a)), as well as lower ability to invade them (Figure 4(b)). These results suggest that CarO is involved in *A. baumannii* adhesion and invasion of human lung epithelial cells.

### Virulence roles of A. baumannii CarO evaluated by a peritoneal sepsis murine model

#### Effect of CarO in the mortality rate induced in mice by A. baumannii cells

**Figure 5.**
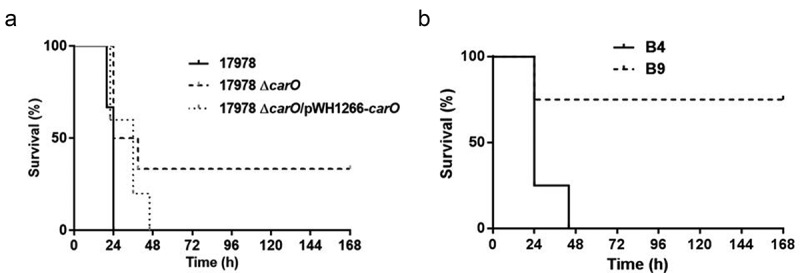
Analysis of mice survival time in the peritoneal sepsis model by (a) 3.20 log_10_ CFU/mL of *A. baumannii* ATCC 17978 wt (17978), its isogenic Δ*carO* mutant (17978 Δ*carO*), and the complemented strain (17978 Δ*carO*/pWH1266-*carO*) (n = 6 mice/strain); and by (b) 4.00 log_10_ CFU/mL of the *A. baumannii* clinical isolates B4 and B9 (n = 4 mice/isolate). Survival curves showed the percentages of mice survival during 7 days. *P* = 0.03 for 17978 *vs*. 17978 Δ*carO*, and *P* = 0.04 for B4 *vs*. B9, log-rank test

Differences in MLD were found between the 17978 wt strain and its isogenic mutant 17978 Δ*carO* (3.20 log_10_ CFU/mL *vs*. 4.30 log_10_ CFU/mL, respectively), and also between the CRAB clinical isolate with a functional CarO protein, B4, *vs*. the one with a truncated CarO, B9 (4.00 log_10_ CFU/mL *vs*. 5.18 log_10_ CFU/mL, respectively). Moreover, survival analyses revealed differences between animals inoculated with the different strains/isolates using the MLD of those with complete *carO* gene (Figure 5). These results indicated that CarO plays an important role in the infective capacity and mortality caused by *A. baumannii in vivo*.

#### Effect of CarO in in vivo dissemination of A. baumannii 17978 cells into mice tissues and fluids

**Figure 6.**
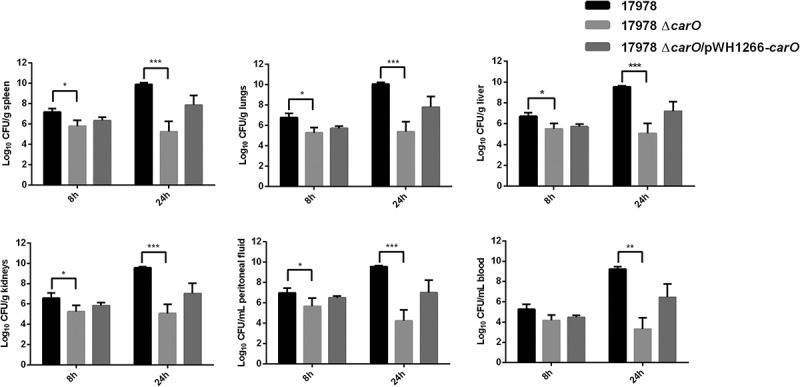
Bacterial burdens in peritoneal sepsis murine model. Bacterial loads in tissues and fluids were determined at 8 h and 24 h after intraperitoneal infection with 3.2 log_10_ CFU/mL of *A. baumannii* ATCC 17978 wild-type (17978), its isogenic *carO* deletion mutant (17978 Δ*carO*), and the Δ*carO* complemented strain (17978 Δ*carO*/pWH1266-*carO*). Data are represented as mean ± SEM (n = 7 mice/strain for each time point). *P < 0.05, ** *P* < 0.01, *** *P* < 0.001, Student’s t-test and Mann-Whitney test

We next compared the dissemination of the 17978 wt, mutant, and complemented strains into different organs and fluids in mice inoculated i.p. with 3.2 log_10_ CFU/mL of each bacterial strain (Figure 6). Mice infected with the 17978 Δ*carO* strain showed at 8 h significant lower bacterial burden in all organs and fluids studied when compared with animals infected with the 17978 wt strain. These differences were even greater 24 h post-infection. Complementation of the Δ*carO* mutant restored almost the wt count levels at 8 and 24 h. These results indicate that CarO affects the dissemination of *A. baumannii* in mice.

## Discussion

In this study, we first investigated the virulence factors of CRAB clinical isolates from six adult patients with BSI secondary to *A. baumannii* VAP. Despite their demographics and clinical characteristics homogeneity, as well as receiving optimal therapy with colistin, three patients survived while the others three did not, despite not having antecedents of other acute and severe infections, or chronic underlying diseases. Using a combination of experimental approaches, we detected that the OMP CarO was present in the isolates from non-surviving patients, while the isolates from the group of surviving patients lacked it due to the selection of different mutations affecting the *carO* gene. Then, additional genetic evidence indicating a virulence role in *A. baumannii* for CarO was obtained by using the model type strains ATCC 17978 and ATCC 19606, their isogenic Δ*carO* mutants, and the Δ*carO* mutants complemented with a plasmid reinstating CarO expression, as well as two of the CRAB clinical isolates. Significant reductions were observed in adherence and invasion of cultured human lung epithelial cells, as well as higher MLD and lower dissemination into essential organs and fluids in the peritoneal sepsis murine model, in strains/isolate lacking CarO.

CarO is the second most abundant protein after OmpA in the *A. baumannii* OM [21] and it has been previously associated with the acquisition of carbapenem resistance (when it is lost or mutated) due to its participation in the selective permeation of imipenem across the *Acinetobacter* OM [21,25,31,32]. Nevertheless, similarly to OmpA [33], several evidences exist suggesting that this protein could also play role(s) in pathogenesis. First, it has been noted that *A. baumannii* can catabolize both arginine and ornithine [32], two basic amino acids present in the blood of mammals in which the arginine-to-ornithine conversion is increased after severe skin injury. This added to the observation that CarO levels in the *A. baumannii* OM are optimum at 37°C and its role as a basic amino acid OM channel, led to suggest that CarO may form part of a fine-tuned mechanism of response to specific signals when confronting a compromised host [32]. Second, four well-defined allelic variants of CarO co-exist in the *A. baumannii* clinical population, and the cognate genes are frequently exchanged between different *A. baumannii* lineages by horizontal gene transfer and assortative recombination; an exchange that could facilitate, among others, *A. baumannii* persistence by evading the host immune response [21]. Third, increased expression of CarO has been described in *A. baumannii* cells during iron-limiting conditions [34] and biofilm formation [35,36], also suggesting roles for CarO in pathogenesis. Fourth, we reported previously that reduced expression of CarO and OprD in a clinical pandrug-resistant *A. baumannii* isolate was concomitant with lower virulence, thus suggesting also roles for these OMPs in this process [37]. Fifth, it has recently been shown using the *A. baumannii* type strain ATCC 19606 that CarO promotes bacterial adhesion and nasal colonization in mice mainly through inhibiting host cell inflammatory immunity responses, again indicating roles for this OMP in pathogenesis [38]. Thus, different lines of evidence and the results of this work point to CarO as an important factor involved in *A. baumannii* recognition and attachment to epithelial cells, in reaching the bloodstream with the potentiality of causing BSI, and also in the invasion of different organs in mammals. Further work is certainly needed to clarify the exact role(s) of this small β-barrel OMP in *A. baumannii* pathogenesis.

OMPs functioning at the interface with the environment constitutes prime candidates for the design of inhibitors aimed to disturb recognition of target cells by the pathogen [39,40]. Most commonly prescribed antibiotics are either bactericidal or bacteriostatic, and the majority work against a broad spectrum of bacteria. There are key approaches that could help to alleviate the problem of antibiotic resistance: first, the development of therapies focused on the specific treatment of infections caused by a single pathogen highly resistant to antimicrobial agents [41]; and second, targeting bacterial virulence factors without inhibiting bacterial growth, which can slow the development of drug resistance by reducing the selective pressure on the bacteria [39,42]. CarO presence is restricted to the OM of *Moraxellaceae* family members, to which the genus *Acinetobacter* belongs [25]. Therefore, an inhibitor targeted toward CarO might provide us with a dedicated therapeutic agent, affecting not only *A. baumannii* but also other pathogenic species of the *Acinetobacter* genus and even the *Moraxellaceae* family. Recently, our group together with Giralt *et al*. developed a series of OmpA inhibitors and tested their effectiveness *in vitro* and *in vivo* in preventing infection by the most prevalent gram-negative bacilli (GNB) in clinical settings including *A. baumannii* [40]. One of these peptides, a cyclic hexapeptide (AOA-2) lacking bactericidal or cytotoxic activities, was able to inhibit GNB adherence to host cells and biofilm formation, thereby preventing the development of infection *in vitro* and in a murine model of peritoneal sepsis [40]. A further study indicated that this OmpA inhibitor was effective in combination with colistin in an experimental model of severe infection with colistin-resistant *A. baumannii* strains [43]. This drug discovery program is considered as an initial stage of the development of a novel class of antimicrobial agents, and in this context, CarO emerges as an attractive target for drug design.

Previous work from the group of one of us [21] disclosed not only the co-existence of four major *carO* gene variants (I to IV) in the *A. baumannii* population, but also their frequent exchange between clinical strains by assortative recombination. These four CarO protein variants show from 80.2% to 83.2% sequence identity between them, with the polymorphisms concentrated mainly in three well-defined protein regions designated VR1, HR2, and HR3 [21]. More recent crystallographic analyses of recombinant CarO variants [31] indicated that, expectedly, the VR1, HR2, and HR3 regions overlap with the exposed (external) loops EL2, EL3, and EL4, respectively, of these proteins. Structural differences at the EL loops are most likely responsible for the differential permeation efficiencies between CarO variants toward the basic amino acid ornithine, with variant I (found in *A. baumannii* ATCC 19606) showing higher ornithine permeability as compared to variant IV (present in *A. baumannii* ATCC 17978) [21]. Thus, and similarly to *A. baumannii* OmpA also exhibiting sequence polymorphisms [44], there are grounds to support the notion that general CarO inhibitors could be also developed against this nosocomial pathogen. As we show in this work different CarO variants, irrespective of their differences at the EL regions [21], participate in the adherence and invasion of human lung epithelial cells (Figure 4). If CarO plays roles in the selective uptake of ornithine through the *A. baumannii* OM, and the EL regions of this OMP participate in the selective recognition of this “substrate” [21,32], compounds resembling ornithine may offer an attractive possibility for the design of inhibitors aimed to block the attachment of this pathogen to host cells.

In summary, the results of the present study, both from the WGS complemented with proteomic and immunoblot analyses of carbapenem-resistant *A. baumannii* clinical isolates causing bloodstream infections and from *in vitro* and *in vivo* model systems, are consistent with the OMP protein CarO forming part of the virulence repertoire of *A. baumannii*. Thus, CarO is suggested as a new target for the development of inhibitors to deal with infections by carbapenem-resistant *A. baumannii*.

## Supplementary Material

Supplemental Material

Supplemental Material

Supplemental Material

Supplemental Material

## Data Availability

The sequences reported in this paper have been deposited in the National Center for Biotechnology BioProject database (accession number PRJNA417465): PKON00000000 (B1), PKOO00000000 (B4), PKOP00000000 (B7), PKOQ00000000 (B8), PKOS00000000 (B9), and PKOR00000000 (B11).
