# Supplementary material for: Virulence role of the outer membrane protein CarO in carbapenem-resistant Acinetobacter baumannii
Source: Virulence. 2020 Dec 10;11(1):1727–37. doi: 10.1080/21505594.2020.1855912 (PMC7733888; doi:10.1080/21505594.2020.1855912)
Supplement: Supplemental Material [file KVIR_A_1855912_SM8051.docx]

**Text S1:**

**Materials and methods**

**Identification of known resistance and virulence mechanisms**

The ResFinder v3.2 web server (<https://cge.cbs.dtu.dk/services/ResFinder/>) [1] of the CGE was used to identify acquired antimicrobial resistance genes in the assembled WGS data from the six CRAB clinical isolates producing bloodstream infections, using a threshold of 100% identity for the genes encoding β-lactamases and 98% identity for all other genes. Moreover, genes with functional annotations including either the term "resistance" or "antibiotic" were considered as resistance genes. Furthermore, point mutations in the quinolone resistance determining regions (QRDRs) of the genes associated with quinolone resistance (*gyrA* and *parC*) were identified in all the isolates comparing the predicted proteins encoded by *gyrA* and *parC* with the deduced amino acid sequences of *gyrA* (UniProt entry: D0CBH9) and *parC* (UniProt entry: D0CB90) from *A. baumannii* ATCC 19606 (ciprofloxacin-susceptible strain), and also with those from *Escherichia coli* K12 (UniProt entries: P0AES4 and P0AFI2, for the deduced amino acid sequences of *gyrA* and *parC*, respectively) in order to define the position of the mutations according to the *E. coli* protein. The BLASTP (protein BLAST, <https://blast.ncbi.nlm.nih.gov/Blast.cgi?PAGE=Proteins>) [2] tool from the National Center for Biotechnology Information (NCBI) was used for the performance of the comparisons. Similarly, point mutations in the conserved regions of *rpoB* gene, related with rifampicin resistance, was detected following the same procedure described for *gyrA* and *parC,* using the deduced amino acid sequences of *rpoB* (UniProt entry: A3M1G3) from *A. baumannii* ATCC 17978 (rifampicin-susceptible strain). Identification of mutations in *pmrAB* and *lpx* genes was not performed due to all isolates were colistin-susceptible. In addition, a combination of ISfinder (http://www-is.biotoul.fr) [3], BLASTN searching on *Acinetobacter* sequences databases [2], and visual inspection was used for a detailed identification of ISs located around *bla*_OXA_ carbapenemase genes on the sequence data derived from the WGS analysis of the six CRAB clinical isolates.

Genes encoding known virulence factors were identified from the WGS data of the clinical isolates using two different strategies. For both strategies, virulence genes described in the MDR *A. baumannii* isolate AB0057 were used for the comparison, being the *A. baumannii* isolate with the highest number of virulence genes contained in the Virulence Factors of Pathogenic Bacteria database (VFDB) (<http://www.mgc.ac.cn/VFs/>) [4]. The first strategy was based on the VFanalyzer option of the VFDB (<http://www.mgc.ac.cn/cgi-bin/VFs/v5/main.cgi?func=VFanalyzer>) [5]. VFanalyzer is an automatic analysis pipeline for a systematic screen of known/potential virulence factors in given complete/draft bacterial genomes. So, draft genomes (contig files) were uploaded and Option 3 was selected ("Upload private data of raw or annotated genome in FASTA or GenBank format"). VFanalyzer performed an automatic prediction of protein-coding genes using GLIMMER3 and then ran the comparison analysis. For the second strategy, amino acid sequences of all the virulence factors of *A. baumannii* isolate AB0057 contained in VFDB were downloaded, and the inferred protein sequences from the six CRAB clinical isolates were compared to them using the standalone version of BLASTP from the NCBI. A protein was related to a virulence factor present in the database when the BLAST hit indicated an identity ≥ 95% for a query coverage ≥ 95%. Thus, using both strategies, the ability to detect all virulence genes were higher than using one alone.

**Growth curves**

The growth profiles of all strains and isolates used for *in vitro* and *in vivo* studies were compared. Thus, 5 × 10^5^ CFU/mL were grown in 10 mL of MHB (with antibiotic when necessary). At 2, 4, 8, and 24 h, the corresponding CFU/mL were determined by plating serial log_10_ dilutions on the corresponding MH agar plates without or with antibiotic, depending on the strain, and on blood agar plates the clinical isolates.

**Text S2:**

**Results**

**Insertion sequences identified around *bla*_OXA_ genes**

Most class D β-lactamases are weak carbapenemases, and overproduction in
*A. baumannii* is generally required to generate the cellular levels compatible with a carbapenem-resistant phenotype [6]. This may result from the antibiotic-mediated selection of a strong promoter derived from the insertion of particular ISs upstream of the *bla*_OXA_ gene [6]. Our analysis of the genomic context of the different *bla*_OXA_ genes detected in the CRAB clinical isolates analyzed here (Supplementary Data set S1) indicated in B1 an IS*Aba1* element inserted 7 bp upstream of the start codon of the chromosomal *bla*_OXA-109_ gene, an arrangement previously shown to largely increase *bla*_OXA_ expression to levels compatible with carbapenem resistance [6]. Most notably, we also located the *bla*_OXA-24_ gene also present in this isolate in a 9,094-bp contig, which shared 99% nucleotide identity in the common region with plasmid pABVA01 (8,963 bp) described in CRAB clinical isolates of Italy [7]. In neither B1 nor the Italian isolates the *bla*_OXA-24_ gene was found linked to an upstream IS element, but transformation of pABVA01 into susceptible *A. baumannii* was found to promote a carbapenem-resistant phenotype [7]. Thus, carbapenem resistance in the B1 isolate most probably results from a combination of an IS*Aba1*-mediated overexpression of its chromosomal *bla*_OXA-109_ gene plus a plasmid-borne *bla*_OXA-24_ contribution. In the other five CRAB clinical isolates analyzed here we detected both *bla*_OXA-58_ and *bla*_OXA-66_ genes (Supplementary Data set S1). The analysis of the genomic context of *bla*_OXA-58_ in these five isolates indicated that this gene was bordered by two oppositely-oriented IS*Aba3* elements, in a commonly-found composite transposon carried by *A. baumannii* plasmids [6]. Overexpression of *bla*_OXA-58_ to levels compatible with carbapenem resistance still requires the additional insertion of another IS in the IS*Aba3* element located upstream of the *bla*_OXA-58_ gene [6]. In this context, we found in all five isolates an IS*18* element in this location, thus generating an arrangement identical to that described in plasmid pABIR responsible of the dissemination of carbapenem resistance among *A. baumannii* strains in Lebanese hospitals [8]. Concerning the chromosomally-encoded *bla*_OXA-66_ also found in these five isolates, no IS insertion was detected upstream of the corresponding genes. Thus, carbapenem resistance results mainly from the IS*18*-mediated overexpression of *bla*_OXA-58_ in all of these cases.

**References in Texts S1 and S2**

1. Zankari E, Hasman H, Cosentino S, Vestergaard M, Rasmussen S, Lund O, Aarestrup FM, Larsen MV. Identification of acquired antimicrobial resistance genes. J Antimicrob Chemother 2012; 67:2640-4.
2. Camacho C, Coulouris G, Avagyan V, Ma N, Papadopoulos J, Bealer K, Madden TL. BLAST+: architecture and applications. BMC Bioinformatics 2009; 10:421.
3. Siguier P, Perochon J, Lestrade L, Mahillon J, M Chandler. ISfinder: the reference centre for bacterial insertion sequences. Nucleic Acids Res 2006;34 (Database issue):D32-6.
4. Chen L, Yang J, Yu J, Yao Z, Sun L, Shen Y, Jin Q*.* VFDB: a reference database for bacterial virulence factors. Nucleic Acids Res 2005; 33:D325-8.
5. Liu B, Zheng D, Jin Q, Chen L, Yang J. VFDB 2019: a comparative pathogenomic platform with an interactive web interface. Nucleic Acids Res 2019; 47(D1):D687-D92.
6. Evans BA, Amyes SG. OXA β-lactamases. Clin Microbiol Rev 2014; 27:241-63.
7. D'Andrea MM, Giani T, D'Arezzo S, Capone A, Petrosillo N, Visca P, Luzzaro F, Rossolini GM. Characterization of pABVA01, a plasmid encoding the OXA-24 carbapenemase from Italian isolates of *Acinetobacter baumannii*. Antimicrob Agents Chemother 2009; 53:3528-33.
8. Zarrilli R, Vitale D, Di Popolo A, Bagattini M, Daoud Z, Khan AU, Afif C, Triassi M. A plasmid-borne *bla*_OXA-58_ gene confers imipenem resistance to *Acinetobacter baumannii* isolates from a Lebanese hospital. Antimicrob Agents Chemother 2008; 52:4115-20.
