# Supplementary material for: Virulence role of the outer membrane protein CarO in carbapenem-resistant Acinetobacter baumannii
Source: Virulence. 2020 Dec 10;11(1):1727–37. doi: 10.1080/21505594.2020.1855912 (PMC7733888; doi:10.1080/21505594.2020.1855912)
Supplement: Supplemental Material [file KVIR_A_1855912_SM7946.zip › Supplementary_FigureS1.pdf]

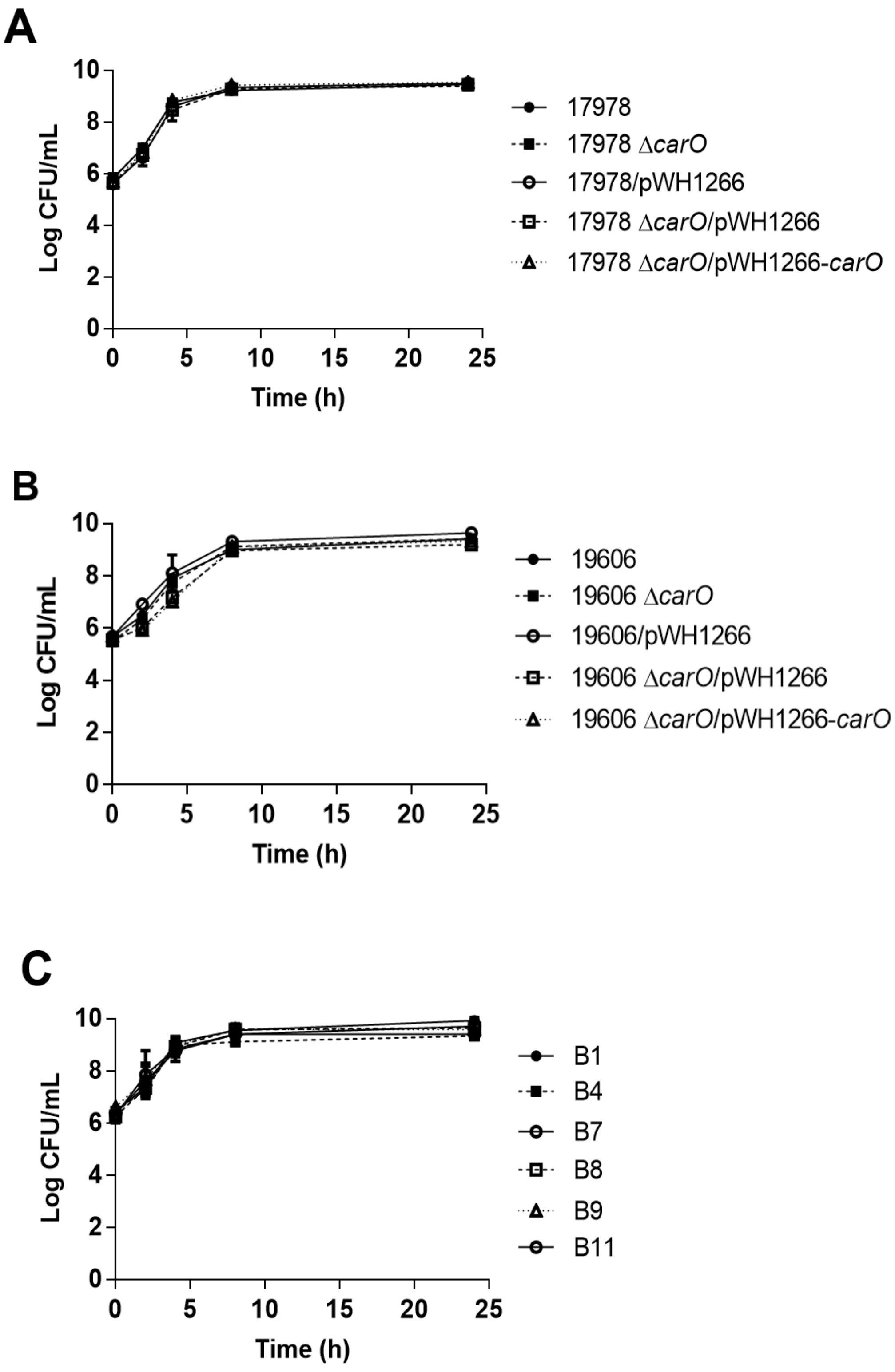

Figure S1. *In vitro* bacterial growth in MHB. All laboratory bacterial strains: *A. baumannii* ATCC 17978 wild-type (17978), 17978 wt with empty plasmid pWH1266 as control (17978/pWH1266), its isogenic *carO* deletion mutant (17978 Δ*carO*), Δ*carO*

mutant with empty plasmid as control (17978 Δ*carO*/pWH1266), and Δ*carO* mutant with plasmid pWH1266-*carO* expressing CarO (17978 Δ*carO*/pWH1266-*carO*) (A); *A. baumannii* ATCC 19606 wild-type (19606), 19606 wt with empty plasmid pWH1266 as control (19606/pWH1266), its isogenic *carO* deletion mutant (19606 Δ*carO*), Δ*carO* mutant with empty plasmid as control (19606 Δ*carO*/pWH1266), and Δ*carO* mutant with plasmid pWH1266-*carO* expressing CarO (19606 Δ*carO*/pWH1266-*carO*) (B); and the six carbapenem-resistant *A. baumannii* clinical isolates from the present study (C), were grown in MHB for 24 h. Data are represented as mean ± SEM (n=3 replicates in different days).
