# Supplementary material for: Virulence role of the outer membrane protein CarO in carbapenem-resistant Acinetobacter baumannii
Source: Virulence. 2020 Dec 10;11(1):1727–37. doi: 10.1080/21505594.2020.1855912 (PMC7733888; doi:10.1080/21505594.2020.1855912)
Supplement: Supplemental Material [file KVIR_A_1855912_SM7946.zip › Supplementary_TableS1.docx]

| **Table S1.** Minimum inhibitory concentrations (MICs) of different antimicrobials for the six CRAB clinical isolates.      **Clinical isolates** | **Imipenem** | **Doripenem** | **Meropenem** | **Ceftazidime** | **Sulbactam** | **Amikacin** | **Tobramycin** | **Ciprofloxacin** | **Rifampicin** | **Doxycycline** | **Tigecycline** | **Colistin** |
| --- | --- | --- | --- | --- | --- | --- | --- | --- | --- | --- | --- | --- |
| **B1** | 64 (R) | > 8 (R) | > 16 (R) | 64 (R) | 32 (R) | 128 (R) | > 128 (R) | 256 (R) | 4 (S) | 16 (R) | 0.5 (S) | 0.06 (S) |
| **B4** | 32 (R) | > 8 (R) | 4 (I) | 128 (R) | 4 (S) | 64 (R) | 128 (R) | 64 (R) | 32 (R) | 32 (R) | 1 (S) | 0.03 (S) |
| **B7** | 8 (R) | 4 (R) | 4 (I) | 128 (R) | 4 (S) | 2 (S) | 128 (R) | 32 (R) | 16 (R) | 32 (R) | 0.5 (S) | 0.06 (S) |
| **B8** | 16 (R) | 4 (R) | 4 (I) | 64 (R) | 4 (S) | 4 (S) | 1 (S) | 16 (R) | 16 (R) | 32 (R) | 0.5 (S) | 0.03 (S) |
| **B9** | 8 (R) | 4 (R) | 4 (I) | 128 (R) | 8 (I) | 64 (R) | 128 (R) | 64 (R) | 32 (R) | 32 (R) | 1 (S) | 0.12 (S) |
| **B11** | 16 (R) | 4 (R) | 4 (I) | 64 (R) | 8 (I) | 2 (S) | > 128 (R) | 16 (R) | 32 (R) | 32 (R) | 0.5 (S) | 0.06 (S) |

^a^S, susceptible; I, intermediate; R, resistant. Breakpoints, imipenem (CLSI): susceptible MIC ≤ 2 µg/mL, intermediate MIC = 4 µg/mL, and resistant MIC ≥ 8 µg/mL; doripenem (CLSI): susceptible MIC ≤ 2 µg/mL, intermediate MIC = 4 µg/mL, and resistant MIC ≥ 8 µg/mL; meropenem (CLSI): susceptible MIC ≤ 2 µg/mL, intermediate MIC = 4 µg/mL, and resistant MIC ≥ 8 µg/mL; ceftazidime (CLSI): susceptible MIC ≤ 8 µg/mL, intermediate MIC = 16 µg/mL, and resistant MIC ≥ 32 µg/mL; sulbactam (CLSI): susceptible MIC ≤ 4 µg/mL, intermediate MIC = 8 µg/mL, and resistant MIC ≥ 16 µg/mL; amikacin (CLSI): susceptible MIC ≤ 16 µg/mL, intermediate MIC = 32 µg/mL, and resistant MIC ≥ 64 µg/mL; tobramycin (CLSI): susceptible MIC ≤ 4 µg/mL, intermediate MIC = 8 µg/mL, and resistant MIC ≥ 16 µg/mL; ciprofloxacin (CLSI): susceptible MIC ≤ 1 µg/mL, intermediate MIC = 2 µg/mL, and resistant MIC ≥ 4 µg/mL; rifampicin (Rev Esp Quimioter 2012;25(2):134-138): susceptible MIC ≤ 4 µg/mL and resistant MIC > 4 µg/mL; doxycycline (CLSI): susceptible MIC ≤ 4 µg/mL, intermediate MIC = 8 µg/mL, and resistant MIC ≥ 16 µg/mL; tigecycline (FDA): susceptible MIC ≤ 2 µg/mL, intermediate MIC = 4 µg/mL, and resistant MIC ≥ 8 µg/mL; colistin (CLSI): susceptible MIC ≤ 2 µg/mL and resistant MIC ≥ 4 µg/mL.

**Antimicrobial agent MIC (µg/mL) (SIR)^a^**
